# Regional Emergence of Water‐Related Browning in a Greening World

**DOI:** 10.1111/gcb.70620

**Published:** 2025-12-11

**Authors:** Rene Orth, Jasper M. C. Denissen, Josephin Kroll, Sungmin O, Ana Bastos, Wantong Li, Diego G. Miralles, Melissa Ruiz‐Vásquez, Anne J. Hoek van Dijke, Andrew F. Feldman, Mirco Migliavacca, Lan Wang‐Erlandsson, Benjamin D. Stocker, Adriaan J. Teuling, Hui Yang, Chunhui Zhan, Xin Yu

**Affiliations:** ^1^ Department of Biogeochemical Integration Max Planck Institute for Biogeochemistry Jena Germany; ^2^ Faculty of Environment and Natural Resources University of Freiburg Freiburg Germany; ^3^ Research Department European Centre for Medium‐Range Weather Forecasts Bonn Germany; ^4^ Department of Electronic and AI System Engineering Kangwon National University Samcheok Republic of Korea; ^5^ Institute for Earth System Science and Remote Sensing Leipzig University Leipzig Germany; ^6^ Department of Environmental Science, Policy and Management UC Berkeley Berkeley California USA; ^7^ Climate and Ecosystem Sciences Division Lawrence Berkeley National Laboratory Berkeley California USA; ^8^ Hydro‐Climate Extremes Lab Ghent University Ghent Belgium; ^9^ Biospheric Sciences Laboratory NASA Goddard Space Flight Center Greenbelt Maryland USA; ^10^ Earth System Science Interdisciplinary Center University of Maryland College Park Maryland USA; ^11^ European Commission Joint Research Centre (JRC) Ispra Italy; ^12^ Stockholm Resilience Centre Stockholm University Stockholm Sweden; ^13^ Bolin Centre for Climate Research Stockholm University Stockholm Sweden; ^14^ Potsdam Institute for Climate Impact Research (PIK) Member of the Leibniz Association Potsdam Germany; ^15^ Institute of Geography University of Bern Bern Switzerland; ^16^ Oeschger Centre for Climate Change Research University of Bern Bern Switzerland; ^17^ Hydrology and Environmental Hydraulics Group Wageningen University & Research Wageningen the Netherlands

**Keywords:** earth observations, earth system models, multiple water variables, regional browning, vegetation greenness, water availability, water limitation

## Abstract

The Earth is greening in many regions under elevated atmospheric CO_2_ concentrations, along with increasing temperature and land use changes. However, despite the continued rise in CO_2_, greening has stagnated or even reversed in some regions, suggesting a reduced capacity of the land surface to act as a carbon sink. Here, we show that declining water availability and rising atmospheric water demand have coincided with regional browning trends over recent decades in some tropical regions that have historically acted as prominent carbon sinks. A regression analysis considering a balanced set of water‐ and energy‐related variables alongside land cover change and climate extremes confirms that both water availability and atmospheric water demand are important contributors to inter‐annual variability in Leaf Area Index (LAI) there. Earth system models mostly reproduce the observed spatial extent of browning and concurrent related coinciding water changes in the multi‐model mean, but results from individual models differ strongly. Our findings provide a new constraint for related model development and highlight the need for enhanced monitoring and consideration of observation‐based water availability trends as an emerging driver of vegetation in future analyses and model development.

## Graphical abstract

The Earth is greening in many regions under elevated atmospheric CO_2_ concentrations, along with increasing temperature and land use changes. However, despite the continued rise in CO_2_, greening has stagnated or even reversed in some regions. Declining water availability and rising atmospheric water demand have coincided with regional browning trends over recent decades in some tropical regions that have historically acted as prominent carbon sinks. Earth system models overall reproduce this while results from individual models differ substantially.

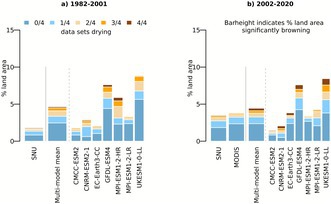

## 1 Introduction

Terrestrial vegetation provides essential ecosystem and climate services such as food supply, carbon storage, and evaporative cooling (Bonan 2008), and contributes to the uptake of about one third of anthropogenic CO_2_ emissions (Gulev et al. 2021; Friedlingstein et al. 2022). Furthermore, vegetation can mediate land surface responses and feedbacks during extreme events such as heat waves (Miralles et al. 2018), droughts (Anderegg et al. 2019), floods (Ukkola et al. 2016; Hoek van Dijke et al. 2022), and fires (O et al. 2020) through its impacts on evaporative cooling (Seneviratne et al. 2010), runoff (Li, Reichstein, et al. 2023), cloud formation (Xu et al. 2022), and precipitation (Smith et al. 2023).

A greening of the Earth's land surface has been observed since the 1980s in many areas across the globe (Donohue et al. 2013; Zhu et al. 2016; Chen et al. 2019; Winkler et al. 2021). This widespread greening has been attributed to multiple factors, including increasing temperature, rising atmospheric CO_2_ concentration (Donohue et al. 2013; Zhu et al. 2016; Winkler et al. 2021) and nitrogen deposition (Zhu et al. 2016), as well as to human land‐use changes and management, e.g. fertilization, irrigation or revegetation (Chen et al. 2019; Ruijsch et al. 2023). The relevance of these drivers varies spatially, where temperature increases are most relevant in high‐latitude regions while management has contributed to greenness trends in India and China (Chen et al. 2019). The greening, in turn, has contributed to changes in ecosystem functioning, such as increased transpiration and associated evaporative cooling (Forzieri et al. 2020; Zhan et al. 2022; Yang et al. 2023), and likely also to an enhanced land sink of carbon (Ruehr et al. 2023). However, greening impacts not only gross CO_2_ uptake but also respiration, with potential compensatory effects that depend on the carbon use efficiency and the disturbance regimes (e.g., Baldocchi 2008). Thus, LAI increases do not necessarily translate directly into stronger carbon sinks.

There is considerable uncertainty on whether the ongoing increases in temperature and CO_2_ (Gonsamo et al. 2021; Gulev et al. 2021) will continue translating into greening (Jiang et al. 2017; Piao et al. 2020; Frankenberg et al. 2021; Winkler et al. 2021), and evidence is mounting for increasing episodic and local browning, which slows down the global greening trend (Feng et al. 2021, Liu, Peng, et al. 2023) in addition to the effects of land use and land cover change. At the same time, the role of water availability and demand is understudied in greening‐related research. Past studies on vegetation greening trends and their drivers have either not separated the effects of vegetation water limitation or found it to be a minor control (Chen et al. 2019; Winkler et al. 2021). Also, in some of these cases, the overall climate change effect is considered without explicitly distinguishing the role of water limitation from that of warming. A search of recent literature in Web of Science (webofscience.com, 2020–2024) using the keywords “vegetation”, “greening”, and “water” or “moisture” reveals that only 32% of the 9440 greening‐related publications explicitly address water‐related aspects. Additionally, while dynamic vegetation models inherently account for water limitation, many of them still struggle to accurately represent their mechanisms and impacts (Gentine et al. 2019; Li, Reichstein, et al. 2023). Water consumption through transpiration is directly linked to CO_2_ assimilation via diffusion through stomatal openings. This way, water is a primary limiting factor for vegetation greenness, particularly in water‐limited ecosystems, in addition to the influence of energy (e.g., temperature, radiation), nutrients (e.g., nitrogen) and CO_2_ (Piao et al. 2019; Denissen et al. 2020). The necessity to account more explicitly for water‐related variables in analyses of long‐term greenness trends is also evident from recent findings on the increasing importance of water availability in controlling monthly vegetation dynamics. For example, enhanced vegetation water limitation on greenness was shown in Feng et al. (2021) and Jiao et al. (2021), while the latter particularly highlights the role of droughts. Also, other recent studies find an increasing sensitivity of vegetation dynamics to soil moisture in many regions across the world, both in recent decades and future projections (Denissen et al. 2022; Li et al. 2022). This increase in the vegetation's sensitivity to water might reflect an increase in water‐limited conditions in response to a changing climate with decreasing soil moisture and increasing vapor pressure deficit projected in many regions (Liu et al. 2020, Gulev et al. 2021). Therefore, water stress can slow down vegetation greening trends by (i) introducing episodic browning where hydraulic vulnerability thresholds are exceeded during days or weeks and the tissue is irreversibly damaged; at the same time (ii) there could also be more gradual changes in LAI by which tree architecture acclimates in response to decadal trends in atmospheric water demand, plant water use efficiency, and water availability.

Considering water‐related variables in vegetation trend analyses is not straightforward. Water availability and atmospheric water demand for vegetation can be represented in different ways using variables such as soil moisture, vapor pressure deficit, precipitation and water potential, or combined metrics such as the aridity index, precipitation minus evaporation, or drought indices. Studies on respective trends yield partly contrasting results (Huang et al. 2016; Vicente‐Serrano, et al. 2022; Liu, Wang, and Yang 2023), probably because the employed indices differ in their consideration of water supply and/or atmospheric water demand. Therefore, multiple water‐related variables should be considered in assessments of vegetation–water interactions in order to capture the underlying complexity.

In this study, we aim to show where and how greenness trends are affected by water‐related variables, and to which extent Earth system models reproduce observed patterns. For this purpose, we compare global patterns of trends in vegetation greenness with those in water availability and atmospheric water demand in observations and models, while also considering other influential variables.

## 2 Data and Methods

In order to study greening and browning trends, we use different Leaf Area Index (LAI) products as indicators of vegetation greenness. We quantify and contrast the trends in annual mean LAI and water‐related variables—including soil moisture, precipitation, dryness index, and vapor pressure deficit (VPD)—during our study period 1982–2020. VPD can decrease vegetation productivity even if soil moisture is not limiting (Fu et al. 2022). In particular, we analyze the agreement of trends across these variables with respect to decreased water availability or increased water demand.

Complementing this trend analysis, we analyze the relationship between inter‐annual dynamics of LAI and water‐related variables in more detail. We perform a regression‐based driver attribution analysis at each grid cell. Therein, we consider an equal number of water‐related and energy‐related variables, as well as land cover change. These include annual averages of (i) tree cover fraction and crop fraction, (ii) the water‐related variables (soil moisture, precipitation, VPD), and (iii) the energy‐related variables (temperature, net radiation, shortwave downward radiation), and additionally, (iv) we use monthly soil moisture minima and daily temperature maxima per year to identify droughts and heat waves. This allows us to compare the impact of gradual changes in hydro‐meteorological variables on vegetation greenness with that of hydro‐meteorological extremes.

The analyses are performed with both observation‐based data and Earth system model output. Observation‐based data include hydro‐meteorological variables sourced from various independent datasets, and LAI data from the Seoul National University (SNU, Jeong et al. 2024) and Moderate Resolution Imaging Spectroradiometer (MODIS, Myneni et al. 2021) datasets. In addition, we consider the same variables from simulations from seven Earth system models from the sixth phase of the Coupled Model Intercomparison Project (CMIP6) which provide all variables required for our analysis.

Finally, we separate our study period into two halves, 1982–2001 and 2002–2020, because this (i) allows detecting emerging signals which may be overshadowed when considering the entire period, and (ii) acknowledges the change in data quality over time, which is particularly relevant in the case of LAI with the start of the MODIS data in 2002 which can therefore be used for validating our results in the second half of the study period.

### 2.1 Observation‐Based Data

An overview of the observation‐based variables and datasets is provided in Table S1. The water‐related variables are sourced from independent datasets. Note, however, that GLEAM soil moisture is forced with MSWEP precipitation data. CPC (Chen et al. 2008) is considered an alternative precipitation dataset to verify our results. Soil moisture is considered across the root zone, where the depth is 250 cm for tall vegetation and 100 cm for short vegetation. VPD is calculated based on the 2 m air temperature and dew point temperature (*T* and *T*_*d*_, respectively) from which we infer the saturation vapor pressure *e*_*s*_ and the actual vapor pressure *e*_*a*_ (both in Pa).

$$e_{s}\left(T\right)=0.6108*\exp\left(\frac{17.27*T}{T+237.3}\right)$$

$$e_{a}\left(T_{d}\right)=0.6108*\exp\left(\frac{17.27*T_{d}}{T_{d}+237.3}\right)$$

$$\mathrm{VPD}=e_{s}\left(T\right)–e_{a}\left(T_{d}\right)$$

Meteorological data (temperature, shortwave radiation, and net radiation) are obtained from the ERA5 reanalysis, (Hersbach et al. 2020). While ERA5 does not include inter‐annual dynamics nor trends in greenness, it is deemed suitable for our analysis thanks to its comprehensive data assimilation across many quantities, which can compensate for some model shortcomings. The dryness index is calculated as the ratio between net radiation from ERA5 and unit‐adjusted precipitation from MSWEP.

For LAI, the state‐of‐the‐art SNU dataset was selected as it is a global LAI dataset that is consistent in terms of explicitly reconciling the effects of satellite orbital drift and satellite changes during the time period 1982–2020 (Jeong et al. 2024). As such, it is less likely to suffer from artifacts due to changes in instrumentation, which have proven to jeopardize the interpretation of greenness over long time scales. In addition, for the more recent considered time period 2002–2020, we also use LAI data from MODIS (MOD15A2H.061, Myneni et al. 2021), where we are not discarding data based on quality flags. Finally, we also consider land cover change in terms of dynamic tree cover fraction and crop fraction in each grid cell, as sourced from the ESA CCI land cover dataset (Defourny et al. 2017). Our analyses are performed at 0.5° × 0.5° spatial resolution and annual temporal resolution. For all datasets with different native resolutions, bilinear interpolation is performed for matching the spatial resolution, and average calculation for matching the temporal resolution.

### 2.2 Earth System Model Output

An overview of the employed Earth system models is provided in Table S2. The same hydrological, meteorological and vegetation‐related variables as in the observation‐based analysis are considered in the model‐based analysis. Also, the considered time period is the same, 1982–2020, using historical and SSP5‐8.5 scenario simulations. Additionally, simulations from the SSP5‐8.5 scenario until the end of the century are considered. All simulations are sourced from the Coupled Model Intercomparison Project Phase 6 (CMIP6) archive (Eyring et al. 2016). Earth system models considered here include CMCC‐ESM2, CNRM‐ESM2‐1, EC‐Earth3‐CC, GFDL‐CM4, MPI‐ESM1‐2‐HR, MPI‐ESM1‐2‐LR, and UKESM1‐0‐LL. We use data from these seven Earth system models because (i) all variables required for this analysis are provided and (ii) the models do not prescribe LAI but actually simulate it. All models account for land use change through e.g. crop expansion, pasture development, and wood harvest in the historical simulations. Similar to the observational analysis, annual means are used. Root‐zone soil moisture is computed as an average of soil moisture per soil layer weighted by the model‐dependent thickness of the respective layer between 0 and 100 cm. All employed variables are aggregated temporally to monthly and spatially to 2° × 2° resolution.

### 2.3 Trend Calculation and Related Assessment of Statistical Significance

Trends are calculated during the first (1981–2000) and second (2001–2020), respectively, halves of our analysis period using the Mann–Kendall trend test. Statistical significance is assessed based on the related *p* value with a threshold of 0.1. This threshold is chosen in order to account for the large natural variability in the considered variables and relatively short annual time series that make it difficult to establish statistical significance. Only in the case of aridity, trends are calculated with an alternative approach because aridity is inherently a long‐term climatic property, unlike the other considered hydro‐meteorological variables or LAI. Estimating it over short temporal windows may introduce random variability and obscure actual climatic trends. We calculate aridity trends by subtracting the mean of the first half of the considered time period from the mean of the second half. The statistical significance of increases or decreases of specific variables in specific time periods is determined through bootstrapping. For this purpose, the difference of the mean values of the first and second half of the considered time period is compared with differences from 300 randomly drawn groups of two samples of the same number of values as the first and second half of the time period, coming from the first‐ and second‐period data samples. If the difference between the values of the first and second half of the time period is larger than the 90th percentile or smaller than the 10th percentile of the differences from the randomly drawn samples, it is considered significant.

### 2.4 Regression Analysis

We evaluate the effectiveness of various predictors to reproduce inter‐annual variations in LAI. The predictors include annual averages of soil moisture, precipitation, vapor pressure deficit, shortwave incoming radiation, net radiation, tree cover fraction, crop cover fraction, and temperature, along with annual minima of monthly soil moisture and daily maxima of temperature. Monthly and daily extreme values from across each year are used here in order to mimic the typical time scales of droughts and heat waves, respectively. The predictors are normalized to range between 0 and 1 by dividing them by their maximum value.

A multiple linear regression framework was employed, where the response variable *y* for each grid cell is modeled as

$$y=\beta_{0}+\beta_{1}x_{1}+\beta_{2}x_{2}+\ldots +\beta_{n}x_{n}+\epsilon$$

where *x*_1_, *x*_2_, …, *x*_*n*_ are the predictor variables, *β*_0_ is the intercept, *β*_*i*_ are the regression coefficients, and ϵ is the residual error term.

In order to address the problem of collinearity between predictor variables, model selection was conducted using the dredge function from the MuMin package (Burnham and Anderson 2004; Bartoń 2025), similar to the methodology used by Fernández‐Martínez et al. (2020) and Denissen et al. (2022). This function tests all possible combinations of predictors and ranks them based on the Akaike information criterion (AIC), allowing us to identify a set of models that balance both performance (likelihood) and complexity (number of predictors). Thereby, collinearity can lead to lower AIC related to overlapping information content between variables such that predictor combinations with a high degree of collinearity will rank lower. We select models whose AIC difference from the top‐ranked model is less than 2, yielding one or more similarly performing models per grid cell. Only models with satisfactory predictive power (adjusted *R* ^2^ > 0.36) are included in the attribution analysis to ensure meaningful interpretability. This threshold is chosen as a compromise between thresholds proposed by Cohen (1988) (0.26) and Moriasi et al. (2015) (0.4).

Based on the selected models in each grid cell, we determine the ranking of the most relevant predictor variables which explain most of the inter‐annual LAI dynamics. If only one model with a single predictor exists, this predictor is considered the most important for that grid cell. When a multivariate model contains multiple predictors, the most influential variable is determined using the variance explained by each predictor, calculated with the ‘lmg’ metric in the relaimpo R package (Groemping 2006), based on the Lindeman–Merenda–Gold method. In cases with multiple multivariate models, the most important predictor is chosen based on the average variance explained across all models, weighted by the Akaike weights.

## 3 Results and Discussion

### 3.1 Global Trends in LAI and Water‐Related Variables

**FIGURE 1:**
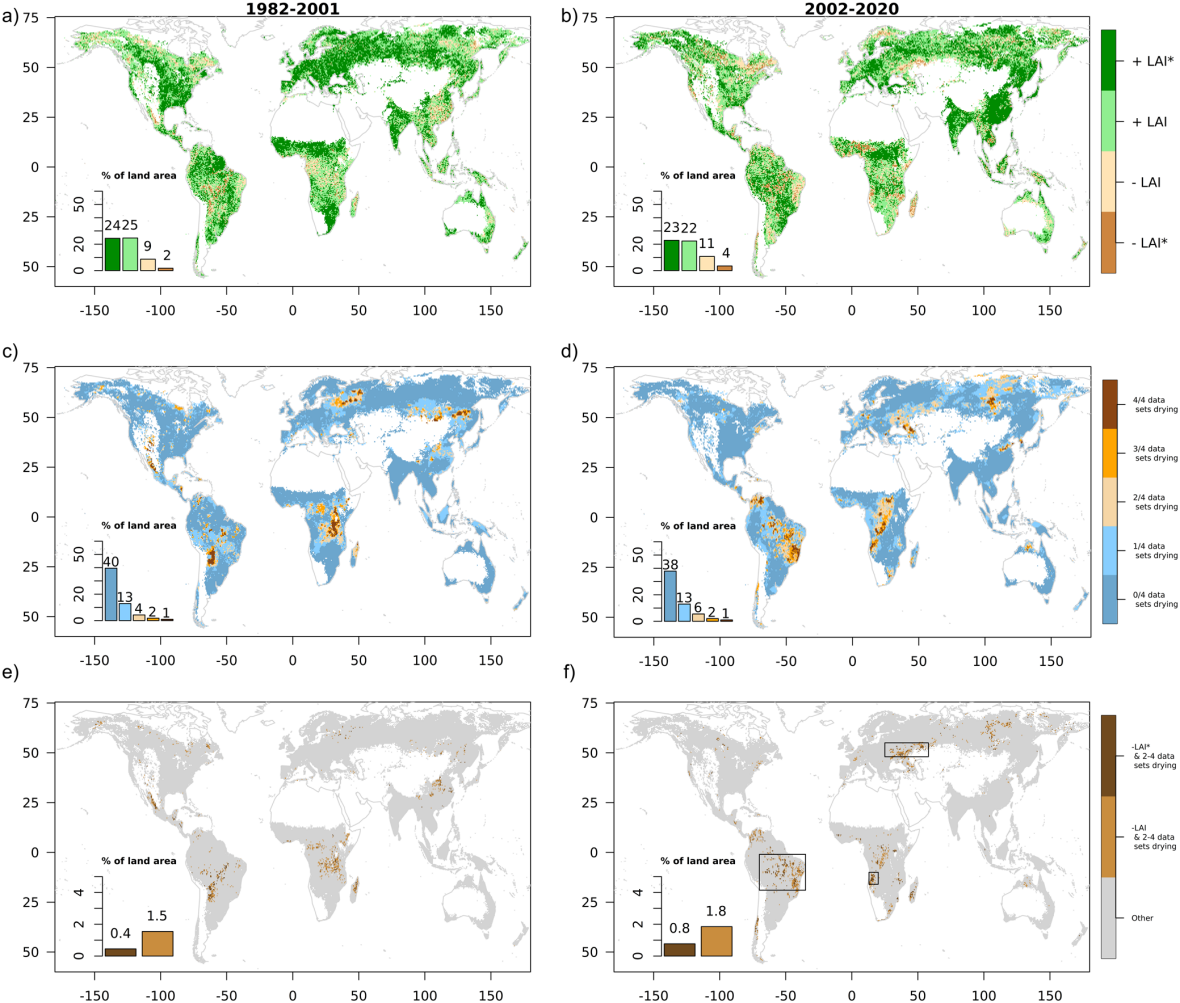
Observation‐based trends in leaf area index (LAI) and water variables during 1982–2001 (left) and 2002–2020 (right). (a, b) Trends of LAI based on SNU data. Significant trends at the 90% confidence level are marked with asterisks (*). (c, d) Agreement of water‐related variables on significant drying trends, defined as decreasing water availability or increasing water demand (90% confidence level); variables considered are root‐zone soil moisture, precipitation, vapor pressure deficit and dryness index (calculated as the ratio between net radiation and precipitation). (e, f) Regions of coinciding browning and drying trends. Boxes in panel (f) denote focus regions. Trends and their significance are determined through the Mann–Kendall test. White areas are excluded from the study area because the decadal mean LAI is below 0.5 in any of the four considered decades. Map lines delineate study areas and do not necessarily depict accepted national boundaries.

Figure 1a,b shows observation‐based evidence for widespread global greening during 1982–2001, particularly in the northern mid and high latitudes and the tropics. However, this greening trend slows down during 2002–2020, with regionalized browning in addition to continued greening in other regions. The browning emerges or intensifies mostly in tropical regions such as the eastern Amazon and western central Africa, as well as in south‐western Eurasia and in the high latitudes across Canada, Scandinavia, and eastern Siberia. This could be related to water‐related variables exerting constraints on vegetation greenness, overruling the influence of radiation and CO_2_ increases. The SNU product employed here aims to compensate for inconsistencies in the AVHRR data, and the results are largely consistent with independent LAI data from MODIS (Figure S1). Additionally, we analyze the differences between the LAI trends between both considered time periods (Figure S2). This confirms the emerging browning in tropical regions and south‐western Russia. Furthermore, it shows reduced greening that did not yet turn into browning in many regions across the tropics and the rest of the globe. Also, intensified greening is observed, for example in eastern Asia and central‐southern South America.

Next, we analyze the trend agreement across four water‐related variables that represent decreased water availability and/or increased water demand. The resulting drying hot‐spot regions are shown in Figure 1c,d. A consistent and significant drying trend with an agreement of drying in at least 2 of the 4 considered water‐related variables is observed for ~7% of the considered land area during 1982–2001, increasing to ~9% during 2002–2020. The drying observed within areas of significant browning is more widespread, with more than 20% in both considered time periods. Note that this considers only regions where drying is statistically significant; numbers would be larger if any drying trends were considered (Figure S3). Interestingly, the regions with strong drying change between the two halves of our study period. During 1982–2001, drying hotspots are found south of the Amazon, in eastern tropical Africa and in north‐eastern Europe, whereas during 2002–2020 drying hotspots are in the eastern Amazon, central tropical Africa and western Russia. Figure S3 displays the trends of the individual water‐related variables. Some similarity is found between the spatial trend patterns of the individual variables, especially between soil moisture and precipitation, but less so for VPD. However, the existence and extent of drying differ in some regions. This highlights the complexity of changes in water availability and demand, which are also related to each other. The largest extent of drying regions is found for VPD and is related to increasing temperatures across most of the globe, allowing the air to hold more water vapor, which consequently leads to decreasing relative humidity and increasing atmospheric water demand. We also analyze the relationship of the spatial patterns of the trends of the individual water variables (Figure S3) with that of LAI (Figure 1a,b) and find weak correspondence (Cramer's *V* around or below 0.1 across water‐related variables and time periods). This suggests that water‐related variables affect LAI mainly jointly, rather than through the dominant influence of individual variables. Note that the statistical significance of the detected trends discussed here is not only affected by the strength of the trends but also by the interannual variability of the variables, where the latter differs across regions.

Finally, to investigate if drying trends potentially contribute to browning trends, we identify regions where drying trends coincide with browning trends in Figure 1e,f. In the first time period (1982–2001) we find relatively few regions with coinciding browning and drying, and the browning is mostly insignificant. This is also related to the fact that there is less total area with browning or drying, respectively, detected during this period. During 2002–2020, however, some larger areas emerge with combined significant browning and drying, mostly across the Amazon, western‐central tropical Africa, and south‐western Russia (see boxes in Figure 1f). The tropical regions are carbon sink hotspots. This suggests that changes in water‐related variables could be an emerging and regionally important driver of vegetation browning, particularly in regions with historically strong land carbon uptake. Note that the extent of the determined regions with coinciding drying and browning is a conservative estimate because water‐related areas may not only induce browning (as considered here) but additionally slow down greening as observed in many additional regions (Figure S2). Moreover, trend results shown here depend to some extent on the chosen year to distinguish between both considered time periods. Finally, given the uncertainty in observation‐based global precipitation datasets, we repeat the analyses in Figure 1 with an alternative precipitation dataset (Figure S4). The results show very similar signals and spatial patterns, suggesting that they are robust with respect to the choice of the precipitation dataset.

**FIGURE 2:**
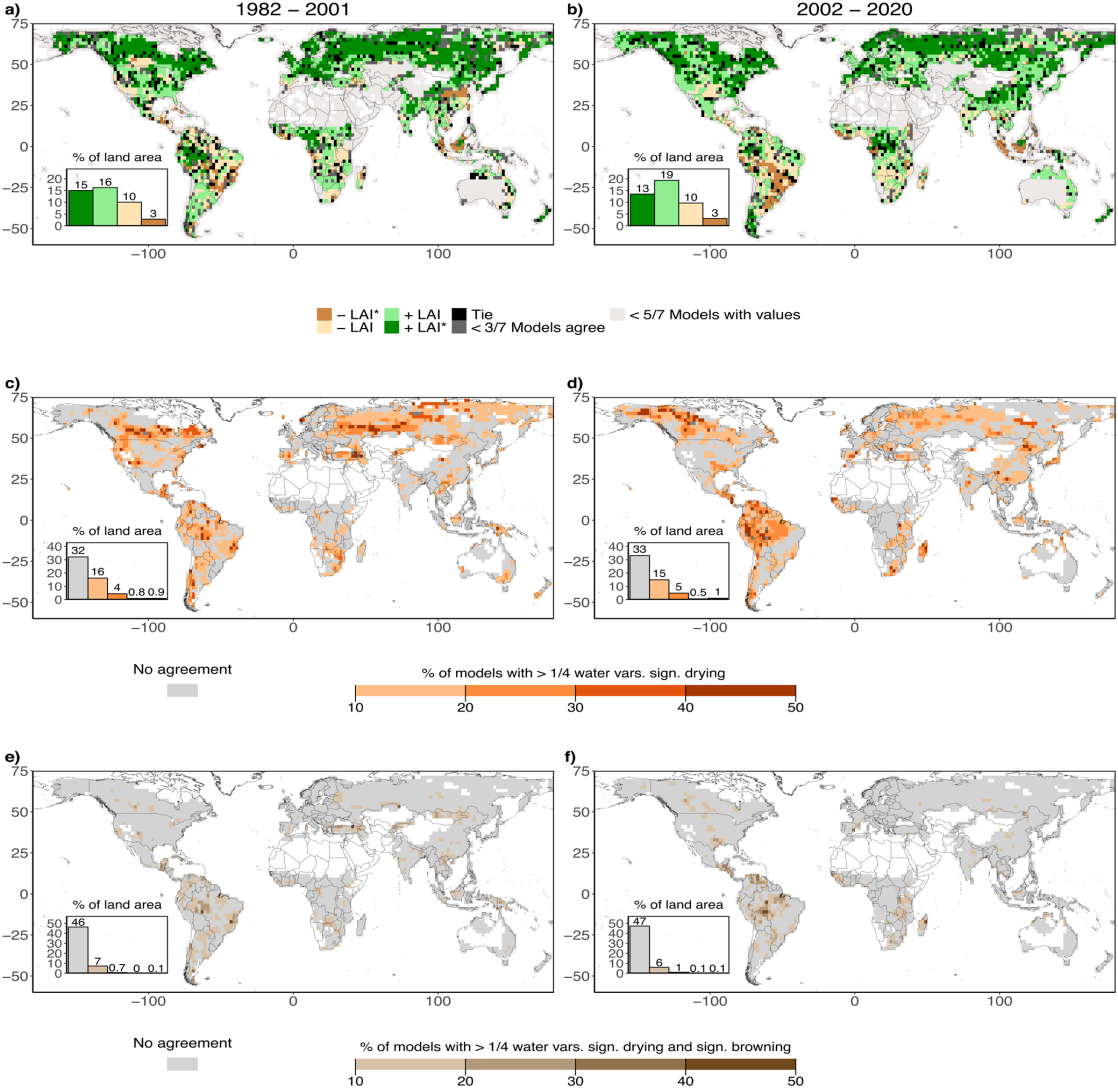
Model‐based trends in leaf area index (LAI) and water variables during 1982–2001 (left) and 2002–2020 (right). Analysis is performed for each of the seven Earth system models separately, and the figure shows the summarized results. (a, b) Trends in LAI. Coloring of each grid cell indicates the most frequent trend category (significant/nonsignificant greening or browning) across the model ensemble. Significance trends at the 90% level are marked with asterisks (*). Gray colors indicate limited model data availability or low agreement between models. Black color denotes that more than one trend category is most occurring across the same number of models. (c, d) Fraction of models simulating significant drying for at least two variables. Drying is quantified through decreasing water availability and/or increasing water demand; variables considered are root‐zone soil moisture, precipitation, vapor pressure deficit, and dryness index. (e, f) Regions of coinciding browning and drying trends. Analyses are restricted to grid cells with data from at least five models. Trends and their significance are determined with a Mann–Kendall test. White areas are excluded from the study area as the decadal mean LAI is below 0.5 in any of the four considered decades. Map lines delineate study areas and do not necessarily depict accepted national boundaries.

Figure 2 investigates modeled trends in LAI and water‐related variables using outputs from seven Earth system models. Historical simulations are used until 2015, and simulations come from the SSP5‐8.5 scenario (O'Neill et al. 2016). Figure 2a,b show that models are overall showing global greening in both time periods, including the large‐scale spatial pattern with the strongest agreement between models on pronounced greening across the northern mid and high latitudes, and relatively weaker greening in tropical regions. Compared with satellite‐based LAI, the models simulate browning over similarly large regions during both considered time periods. However, the extent of (significant and nonsignificant) browning slightly decreases in the model results, while it increases in the observation‐based data. It remains unclear whether this indicates model shortcomings, whether it is related to the temporal inconsistencies underlying the observation‐based LAI data, or both. There are considerable differences between the results of individual models as shown in Figures S5 and S6; while greening prevails for all of them, the extent of browning (as well as its spatial patterns) largely differs across models. Models with least and most widespread browning also vary between the time periods.

Figure 2c,d displays the dryness trends simulated by the models shown through the agreement of models on significant drying in terms of at least half of the considered water‐related variables (precipitation, soil moisture, dryness index, and vapor pressure deficit, as for the observation‐based analysis). While models agree that drying is happening, they do not agree well on the related regions. Typically, not even a third of the models agree on regions of drying. Regions with the strongest agreement are scattered across the globe and mostly found across the high northern latitudes, the Amazon and southeastern Africa in both time periods. Figure 2e,f shows the model agreement in terms of areas with coinciding browning and drying. Model agreement is very low with only a few areas scattered across the tropics showing coinciding browning and drying in more than one model. Also, when considering areas with nonsignificant browning, model agreement is only slightly higher (Figure S7).

### 3.2 Trends of Water Availability and Demand in Browning Regions

**FIGURE 3:**
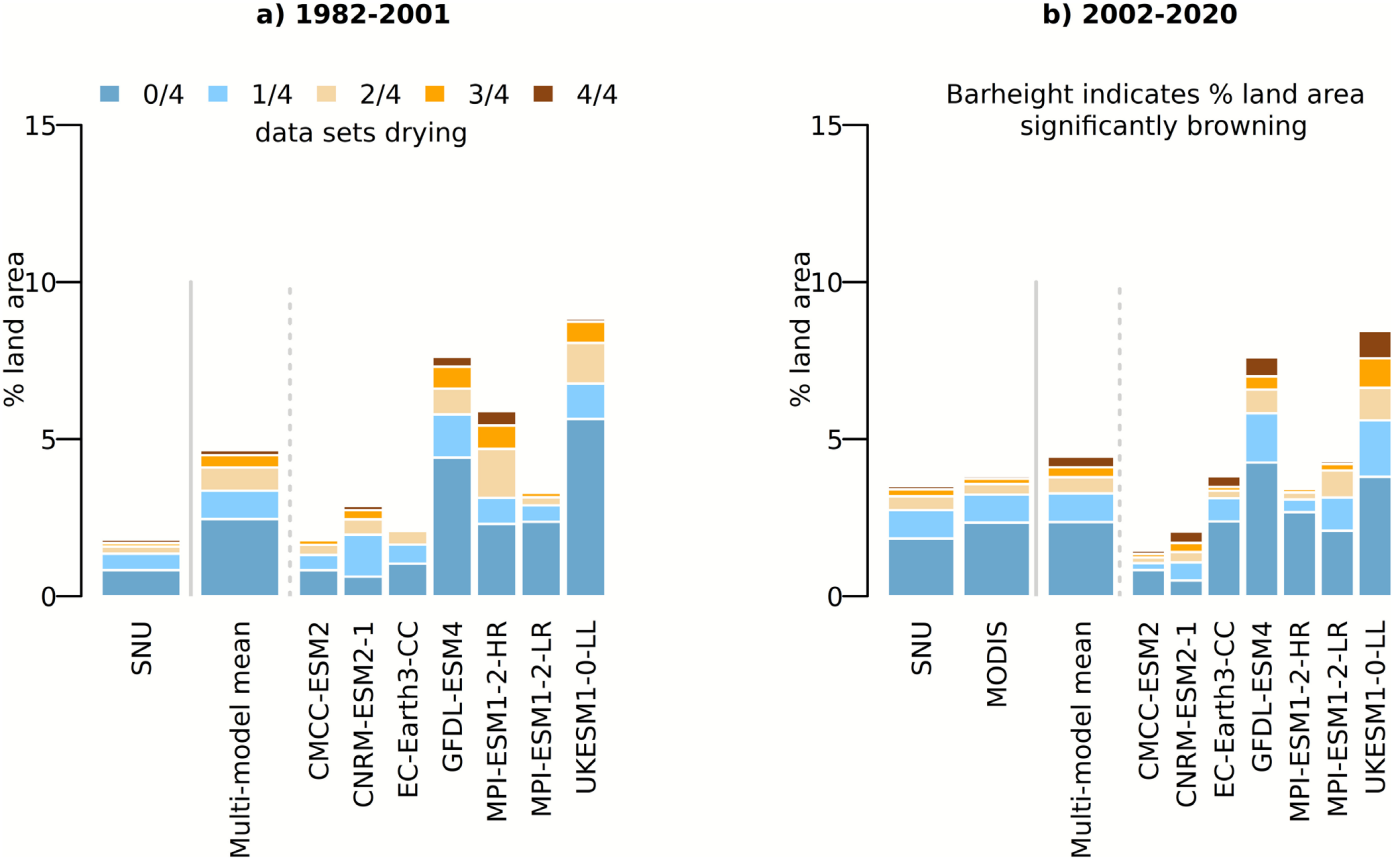
Degree of drying in areas of significant browning in observations and models during (a) 1982–2001 and (b) 2002–2020. Height of bars indicates the size of the area with significant browning. Bar colors denote the agreement of water‐related datasets on drying, quantified by decreasing water availability or increasing water demand; variables considered are root‐zone soil moisture, precipitation, vapor pressure deficit and dryness index. Observation‐based results from MODIS shown for the 2002–2020 period only due to limited data availability. Multi‐model mean is calculated as the average across results from individual models.

In the next step, we focus explicitly on browning regions and analyze the degree of drying found within these regions. Figure 3a confirms that models simulate a larger extent of significant browning during 1982–2001 compared to observations. In both the observation‐based and the model‐based results, drying as detected in at least half of the considered water variables is only found in a small fraction of the browning areas. These results indicate that the browning may be related to water‐related constraints regionally, while other causes seem to prevail in most browning regions. These include human impacts through land management and land use changes (Carvalho et al. 2019; Chen et al. 2019; Liu et al. 2021). The results across individual models vary considerably, both in terms of the simulated extent of browning (as also seen in Figures S5 and S6) and the simulated extent of coinciding drying. This is an important result and is probably related to shortcomings in the representation of vegetation in the models. For example, (i) greenness can also change at longer time scales due to changes in vegetation composition as a result of natural succession and ecological dynamics, e.g., grasses are replaced by shrubs or a young forest becomes a mature forest and (ii) vegetation can also cope with drying trends through, for example, increased atmospheric CO_2_ concentration which can contribute to enhanced intrinsic plant water use efficiency (Gonsamo et al. 2021; Zhang et al. 2022; Jiao et al. 2021; Vicente‐Serrano, Miralles, et al. 2022), and by reducing their water limitation by structural adaptation through for example deeper roots (Fan et al. 2017; Singh et al. 2022; Stocker et al. 2023). These mechanisms tend to reduce vegetation sensitivity to water (Stocker et al. 2023).

Figure 3b shows similar model‐based results for the period 2002–2020, even though individual models simulate slightly different extents of browning and drying compared with the earlier time period. Further, the multi‐model mean results are similar to the observation‐based results for this time period with regard to the extent of browning as well as the coinciding drying. We reproduce Figure 3, additionally including regions of insignificant browning (Figure S8). This way, we find clearly more regions with coinciding browning and drying across the globe. This suggests that natural variability in LAI and water‐related variables, which limits the statistical significance in our analysis in Figures 1 and 2, is masking some of the relevance of water‐related variables for vegetation greenness.

Moreover, Figure 3 confirms observation‐based results from Figure 1 of increased browning in observations between the considered time periods, and of increased coincidence of browning and drying. This is in line with previous studies providing evidence that vegetation sensitivity to water availability is increasing, which, however, focused on vegetation dynamics at shorter time scales and on individual water‐related variables or indices. For example, Jiao et al. (2021) found an increasing area within the Northern Hemisphere extratropics where NDVI is significantly correlated with water availability as represented through the Standardized Precipitation‐Evaporation Index (SPEI) and the Palmer Drought Severity Index (PDSI). Using a different methodological approach and datasets, Li et al. (2022) found an increasing trend in the global sensitivity of LAI to soil moisture during recent decades. Both studies report that temperature increases play a major role in driving the increased vegetation water sensitivity, in addition to other aspects such as decreasing precipitation, decreasing soil moisture, and changes in vegetation structure and physiology (Zhang et al. 2019). The relevance of each factor varies between regions. While the relevance of precipitation and soil moisture is straightforward, the prominent role of temperature can be explained by its role in increased atmospheric dryness.

We extend this analysis of browning regions and their dryness trends for future time periods using the multi‐model mean (Figure S9). There is no systematic trend in the extent of browning areas during this century, but drying becomes more common in the detected browning areas, and the fraction of browning areas with no associated drying trend decreases.

### 3.3 Drivers of Inter‐Annual LAI Dynamics

**FIGURE 4:**
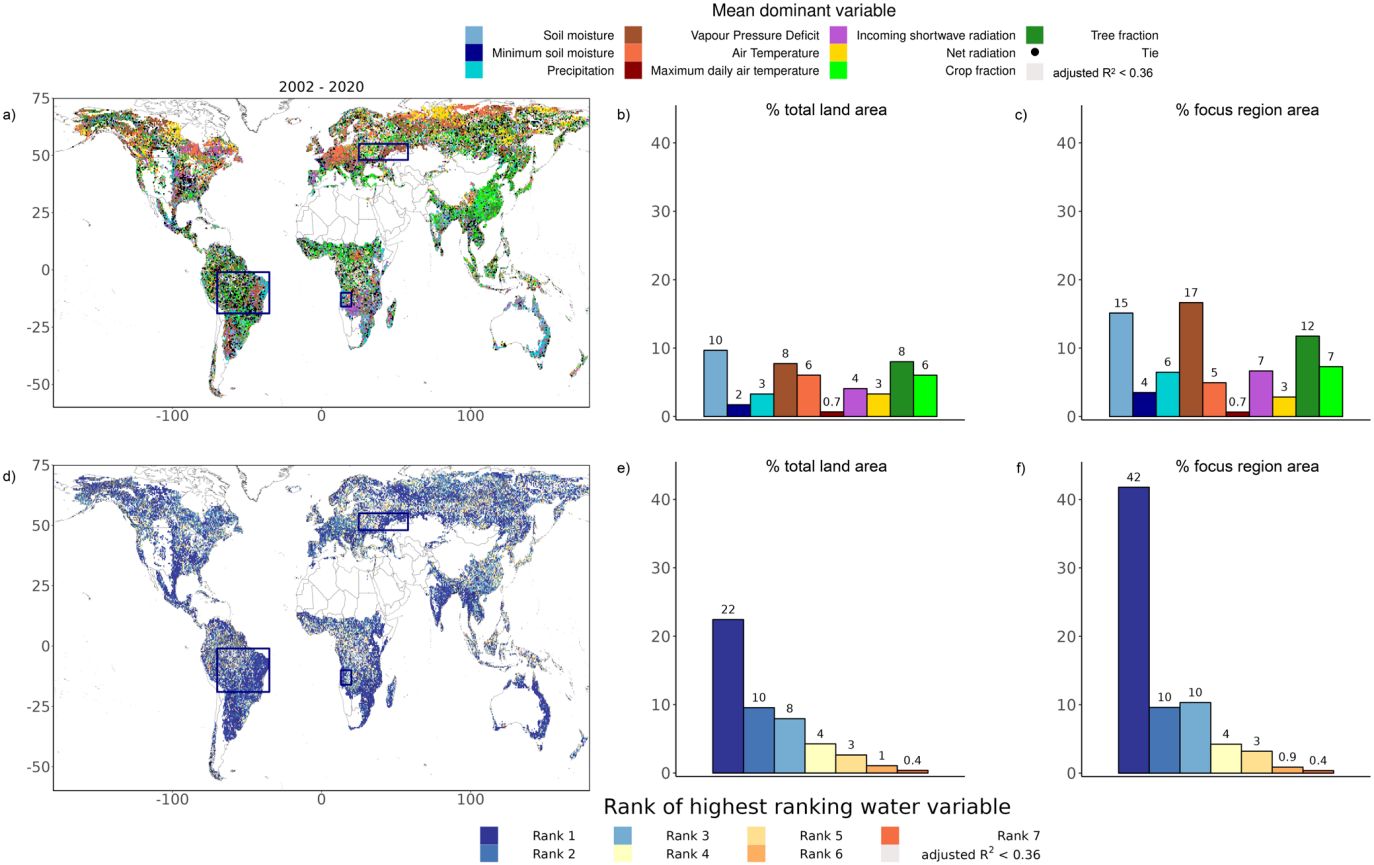
Drivers of inter‐annual LAI dynamics in observation‐based data. (a) Dominant variables as determined in regression‐based analysis during 2002–2020. Black dots indicate that several variables are equally relevant. (b, c) Area coverage where considered variables are found most relevant for LAI dynamics, (b) globally and (c) across the focus regions. (d–f) Similar to (a–c) but for illustrating the highest‐ranked water variable in the regression‐based analysis. Boxes in (a) and (d) denote focus regions identified in Figure 1. Map lines delineate study areas and do not necessarily depict accepted national boundaries.

While we detect regions with coinciding browning and drying trends, spatial overlap does not prove causality. In order to further corroborate the detected coincidence of browning and drying trends, we perform a regression‐based analysis to determine the covariation with the considered water‐related variables, and other environmental variables in explaining inter‐annual LAI dynamics. The importance of variables here is inferred from the Lindeman–Merenda–Gold (LMG) method which partitions the explained variance among predictors in multivariate regression models (see Subsection 2.4). The observation‐based results are shown in Figure 4. The most relevant variable for explaining LAI dynamics varies considerably between regions. All considered variables are most relevant in some region across the globe. Soil moisture, VPD and tree cover change are most relevant across larger areas than other variables. The spatial heterogeneity of the patterns found here suggests that this is modulated by soil and vegetation types, which are similarly heterogeneous in space. Despite this heterogeneity, larger‐scale patterns of prevailing land cover change control LAI dynamics are found in east Asia and across regions in the tropics, confirming earlier findings by Chen et al. (2019). Changes in forest cover are more relevant than changes in crop fraction in affecting LAI which makes sense given the greater leaf area of trees. Furthermore, energy control of LAI dynamics in cold and tropical regions and water control in semiarid regions emerge. These patterns have long been recognized (Budyko 1974) and further investigated more recently (Seneviratne et al. 2010; Forzieri et al. 2017; Papagiannopoulou et al. 2017; Denissen et al. 2020; Fu et al. 2021; Jiao et al. 2021; Feldman et al. 2022; Wang et al. 2022). Many of these studies, however, focused solely on water availability (soil moisture) and did not include atmospheric water demand (VPD) while our results show that both supply‐ and demand‐related water variables are globally relevant and jointly affect LAI dynamics. The spatial extent of the area where water‐related variables are found most relevant for LAI dynamics in Figure 4 is higher in the focus regions with coinciding browning and drying than across the entire global study area, particularly in the case of VPD. Also tree cover change plays an important role in the focus regions, suggesting that deforestation drives LAI dynamics in some places (Chen et al. 2019).

The lower panels in Figure 4 show the inferred rank of the most relevant water‐related variable in explaining LAI dynamics compared with the other considered variables. Also in regions where energy‐related variables are most relevant, water variables can additionally play an important role, as evidenced by water variables ranking second or third in some of these regions. In the eastern Amazon, western tropical Africa and south‐western Russia, where we identified coinciding browning and drying (Figure 1f), water‐related variables are largely found to be most relevant in explaining LAI dynamics. Therefore, these results serve as additional evidence of the regional role of drying in inducing vegetation browning in these regions.

The explanatory power of the regression models tends to be the highest in the subtropics, Europe, and Central America (Figure S10). The explained fraction of variance (*R* ^2^) exceeds our threshold of 0.36 in most regions, while still a substantial fraction of the significance can not be explained by our regression models. Additional variables which are not considered here, such as nutrient availability, changing atmospheric CO_2_ concentration, or snow and ice dynamics, could explain part of the variance in LAI dynamics. More generally, the response of vegetation to concurrent changes in water availability, other climate drivers, and changing CO_2_ concentrations is complex (Brodribb et al. 2020; Walker et al. 2020; Zhan et al. 2022). For example, increasing CO_2_ concentrations can enhance photosynthesis and/or water use efficiency which can consequently affect LAI dynamics. Thereby, the effect of different drivers can also amplify or compensate for each other (De Kauwe et al. 2021; Liu, Wang, and Yang 2023). Also variables can interact with each other such that for example reduced water availability at the land surface can lead to reduced evaporation, which can, in turn, yield increased VPD making it more likely to be detected as a main driver of LAI dynamics (Novick et al. 2016; Fu et al. 2022).

In addition to annual means of water and energy‐related variables, the regression analysis also considers the role of potential extreme events such as soil moisture droughts and heat waves for LAI dynamics. Thereby, potential soil moisture droughts are represented through the minimum monthly soil moisture in each year, and potential heat waves are represented through the daily maximum temperature of the year in each grid cell. Figure 4a–c shows that these variables are less relevant than annual average energy and water conditions. This can be related to the fact that actual extremes only occur in a few years while in most years there are no outstanding temperature maxima or soil moisture minima. However, extremes do matter in specific locations scattered across the globe, and the extent of regions where soil moisture droughts are most relevant is higher in the focus regions than across the entire global study area. Further, previous studies have shown that hydroclimatic extremes including droughts, floods and heat waves can have large impacts on vegetation (Reichstein et al. 2013; Kroll et al. 2022), even though they did not explicitly compare their effect with that of the variability in annual mean hydro‐meteorological conditions. Extreme event impacts can be abrupt, persistent, and difficult or impossible to reverse, due to the crossing of critical thresholds and triggering of regime shifts (Berdugo et al. 2022). The impacts of hydroclimatic extremes on vegetation are induced through multiple biophysical and biochemical processes. For example, in the case of drought (Allen et al. 2015) (i) plants may close their stomata to save water which can lead to carbon starvation or (ii) plants do not close their stomata to fully benefit from the surplus in available radiation, but then risk hydraulic failure (Anderegg et al. 2015; Ruehr et al. 2019). Next to such direct effects, hydroclimatic extremes also introduce cascading effects such as pathogens and insect outbreaks that cause tree mortality (Allen et al. 2015; De Brito 2021). Furthermore, droughts and heatwaves can self‐propagate to neighboring regions—and so can their ecosystem impacts—when reduced evaporation leads to reduced precipitation and higher temperatures (Schumacher et al. 2019, 2022). More frequent extreme weather events in a warming climate (particularly droughts and heat waves, Seneviratne et al. 2021) are likely to affect trends, or to even induce abrupt and lasting shifts in vegetation greenness (He et al. 2023). This can happen as more frequent extreme events might affect the vulnerability of vegetation to future climate change (Anderegg et al. 2020; Forzieri et al. 2022).

**FIGURE 5:**
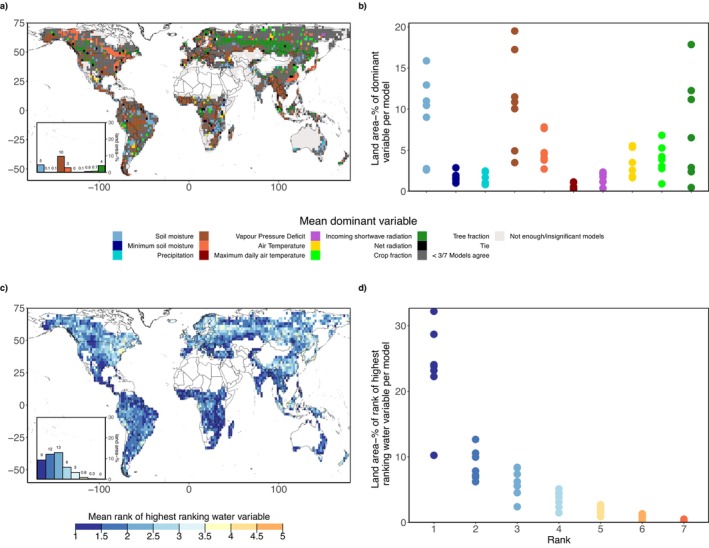
Drivers of inter‐annual LAI dynamics in Earth system models. Analysis is performed for each of seven Earth system models separately, and the figure shows summarized results. (a) Dominant variables as determined in regression‐based analysis during 2002–2020. Dark gray color indicates that less than three models agree on the most‐occurring variable. Black color denotes that different dominant variables are most‐occurring across the same number of models (denoted as Tie). (b) Area coverage where considered variables are found most relevant for LAI dynamics in individual models. (c, d) Similar to (a, b) but for mean rank of the highest‐ranked water variable across models in the regression‐based analysis. Map lines delineate study areas and do not necessarily depict accepted national boundaries.

The regression‐based analysis is also performed with the Earth system model outputs. Figure 5 shows that the models overall agree that soil moisture, VPD and tree cover change are the most relevant variables for LAI dynamics globally. At the same time, the relevance of VPD is larger than in the observation‐based results, while that of soil moisture is smaller. This is related to the fact that most models simulate stomatal conductance which links vegetation functioning to water, based on the Ball‐Berry approach which does not consider soil moisture (Ball et al. 1987). Also in the models, water‐related variables do play a (secondary) role even in energy‐controlled regions as shown in the ranks in Figure 5c,d. There is less agreement between models and observations in terms of the spatial patterns of controlling variables in Figure 5a,b, and in terms of the results for the other considered hydro‐meteorological variables which are found less relevant. However, as Figure 5a,c summarizes results across models it may provide biased results for hydro‐meteorological variables which are only relevant in small and/or scattered regions. Some individual models show that locally, soil moisture droughts are most relevant for LAI dynamics, underlining the importance of extremes versus mean soil moisture conditions (Figure S11). Overall, the main drivers of LAI dynamics differ greatly between models, also in terms of the general relevance of water‐related variables, energy‐related variables, and land cover change, respectively. This is probably related to different representations of the vegetation‐climate coupling in general and the vegetation‐water coupling in particular. Also, the mechanisms through which heat waves and droughts can affect vegetation, including legacy effects, are not fully implemented in models. The models do, however, agree with observations in terms of the spatially multifaceted main controls of LAI dynamics. The explanatory power of the regression models is higher in the analysis of the Earth system model outputs (Figure S12) compared to the observation‐based analysis (Figure S10) which is related to less or no noise in the model simulations given their deterministic nature. In both analyses, *R* ^2^ is lower in the high latitudes, probably due to the confounding influence of snow and ice dynamics on the results.

### 3.4 Consideration of Multiple Water‐Related Variables

Overall, moving beyond previous studies, we incorporate a comprehensive suite of water variables, covering both water availability and demand. All of them are found to be relevant, even though partly in different regions. Including multiple water‐related variables allows us to acknowledge the differences in trends of the individual water‐related variables, to detect regions and time periods where most water‐related variables agree on drying, and to analyze to which extent models acknowledge the influence of this diversity of water‐related variables. Also, the regression‐based analysis suggests that different water‐related variables are relevant for LAI dynamics in different regions such that ignoring their variety would lead to biased results. Another novel aspect of the regression‐based analysis is the joint consideration of the role of means and extremes of soil moisture and temperature for LAI dynamics which shows that means are overall more relevant while extremes matter locally.

In addition to the direct effects of water‐related variables on greenness, vegetation changes can also feed back into the climate system and the water cycle in particular. For example, reduced plant transpiration as a response to water stress translates into changes in runoff (Li, Reichstein, et al. 2023) and precipitation (Hoek van Dijke et al. 2022). Vegetation growth leads to decreased terrestrial water storage in drylands (Liu, Li, et al. 2023), and vegetation greening leads to an increased ratio of transpiration‐to‐evaporation such that surface energy and water fluxes become more controlled by vegetation (Forzieri et al. 2020). Furthermore, greenness influences surface albedo, which is key for the energy balance of the Earth and thereby influences climate (Forzieri et al. 2017; Duveiller et al. 2018; Li, Li, et al. 2023). Other studies show that vegetation affects climate sensitivity, i.e., the response of global mean temperature to increasing atmospheric CO_2_ (Zarakas et al. 2020; He et al. 2022).

## 4 Conclusions

In this study, we highlight the relevance of water availability and demand for vegetation greenness trends by jointly analyzing a comprehensive set of climate and vegetation data streams. We show that water‐related variables can regionally affect greenness; this is found consistently in an analysis of coinciding trend signals and an independent regression analysis on controls of annual LAI dynamics. In fact, vegetation has mechanisms to adapt to changes in water availability and demand such as stomatal regulation or changes in carbon allocation to adjust the rooting depth. The fact that we nevertheless find impacts of drying on vegetation greenness that are not prevented by acclimation, suggests that the changes in water‐related variables are happening more rapidly than the rate at which vegetation can naturally adapt and acclimate to it. Future research could revisit our analysis to focus more specifically on this aspect. Taken together, this evidence highlights that water‐related variables can and should be explicitly considered in analyses of global or regional greening or browning. Importantly, our results likely represent a conservative estimate of the relevance of water‐related variables as we focus only on areas of significant browning while changes in water availability and atmospheric water demand may also slow down greening in some areas that are not (yet) browning. Moreover, soil water availability and atmospheric water demand are only two among many relevant drivers of vegetation greenness, and our results do not question the dominant role of other factors, such as land use change or CO_2_ fertilization.

We perform the analyses in this study for both observation‐based data and Earth system model outputs, enabling direct comparison. Earth system models generally capture the broad relevance of water‐related variables for inter‐annual greenness dynamics. However, while they reproduce these large‐scale relationships, we also identify some disagreements with observation‐basedresults that can inform directions for model development. Specifically, models tend to (i) overestimate the influence of water demand on inter‐annual greenness dynamics, (ii) underestimate global greening during 1982–2001, (iii) underestimate the diversity of influential water‐related variables across different regions, and (iv) display large variation across individual models such that individual models typically show larger disagreement with observation‐based findings than the multi‐model mean. To address these shortcomings, incorporating a more detailed representation of plant hydraulics offers a promising avenue for model development. This could be done for example through the inclusion of more accurate water stress formulations to determine the response of stomatal conductance to variations in soil moisture and atmospheric water demand (e.g., Migliavacca et al. 2021). Such development can in turn help to decrease the considerable spread across models and their future projections. Thereby, accurately capturing the effects of water‐related variables on vegetation trends can provide more precise information for managing water resources and ecosystems under global change.

## Author Contributions

**Rene Orth:** conceptualization, methodology, visualization, writing – original draft, writing – review and editing. **Jasper M. C. Denissen:** conceptualization, formal analysis, methodology, visualization, writing – review and editing. **Josephin Kroll:** conceptualization, formal analysis, methodology, visualization, writing – review and editing. **Sungmin O:** conceptualization, methodology, visualization, writing – review and editing. **Ana Bastos:** methodology, writing – review and editing. **Wantong Li:** methodology, writing – review and editing. **Diego G. Miralles:** methodology, writing – review and editing. **Melissa Ruiz‐Vásquez:** methodology, writing – review and editing. **Anne J. Hoek van Dijke:** methodology, writing – review and editing. **Andrew F. Feldman:** methodology, writing – review and editing. **Mirco Migliavacca:** methodology, writing – review and editing. **Lan Wang‐Erlandsson:** methodology, writing – review and editing. **Benjamin D. Stocker:** methodology, writing – review and editing. **Adriaan J. Teuling:** methodology, writing – review and editing. **Hui Yang:** methodology, writing – review and editing. **Chunhui Zhan:** methodology, writing – review and editing. **Xin Yu:** methodology, writing – review and editing.

## Conflicts of Interest

The authors declare no conflicts of interest.

## Supporting information


**Data S1:** Supporting Information.

## Data Availability

The scripts of the programming code for all analyses and for creating the figures are available at https://doi.org/10.5281/zenodo.17472614. This archive also includes processed data used within our analyses. All employed observation‐based datasets are summarized in Table S1, including related references. Similar information for employed Earth system model simulations is summarized in Table S2. Earth system model simulations were sourced from the Earth System Grid Federation (ESGF) at https://aims2.llnl.gov/search/?project=CMIP6/.
