# Supplementary material for: Regional Emergence of Water‐Related Browning in a Greening World
Source: Glob Chang Biol. 2025 Dec 11;31(12):e70620. doi: 10.1111/gcb.70620 (PMC12696614; doi:10.1111/gcb.70620)
Supplement: Supplementary file 1 — Data S1: Supporting Information. [file GCB-31-e70620-s001.pdf]

Supplementary Material:

Table S1: Overview of employed variables and related datasets. Italic font indicates additional datasets used for validation of the results.

Variable	Data set	Native temporal resolution	Native spatial resolution	Interpolation method	Reference
LAI	SNU	monthly	0.05°	bilinear	Jeong et al. 2024
LAI	*MODIS, MOD15A2H.061*	*8 daily*	*500m*	*bilinear*	*Myneni et al. 2021*
Precipitation	MSWEP v2.8	daily	0.1°	bilinear	Beck et al. 2019
Precipitation	*CPC*	*daily*	*0.5°*	*none*	*Chen et al. 2008*
Soil moisture	GLEAM v4.1	daily	0.1°	bilinear	Miralles et al. 2025
Maximum and mean temperature	ERA5	daily	0.25°	bilinear	Hersbach et al. 2020
Net radiation	ERA5	daily	0.25°	bilinear	Hersbach et al. 2020
Shortwave incoming radiation	ERA5	daily	0.25°	bilinear	Hersbach et al. 2020
Tree and crop cover fraction	ESA-CCI land cover v2	annual	300m	bilinear	Defourny et al. 2017

Table S2: Overview of employed Earth System Models and related information. For all models, the following variables have been extracted at the monthly timescale, according to the CMIP6 nomenclature: temperature (tas), relative humidity (hurs), the total water content per soil layer (mrsol), precipitation (pr), leaf area index (lai), maximum daily temperature (tasmax), incoming and outgoing short- and longwave radiation (rsds, rsus, rlds, and rlus), crop fraction (cropFrac) and tree fraction (treeFrac).

Model	Institution	Member	Version	Irrigation	Land Use Change	Reference
CMCC-ESM2	Centro Euro-	r1i1p1f1	20210114; 20210126	no	yes	Cherchi et al. (2019),

	Mediterraneo sui Cambiamenti Climatici (CMCC)					Lovato and Peano (2020a, b)
CNRM-ESM2-1	Centre National de Recherches Météorologiques	r1i1p1f2	20181206; 20191021	No	yes	Seferian (2018), Seferian et al. (2019), Voldoire (2019)
EC-Earth3-CC	EC-Earth-Consortium	r1i1p1f1	v20210113; v20210113	Indirectly, through irrigated crop	yes	EC-Earth Consortium (2021a,b), Döscher et al. (2022)
GFDL-ESM4	National Oceanic and Atmospheric Administration & Geophysical Fluid Dynamics Laboratory	r1i1p1f1	v20190726; v20180701	no	yes	Dunne et al. (2020), John et al. (2018), Krasting et al. (2018)
MPI-ESM1-2-HR	Max-Planck-institute for Meteorology	r1i1p1f1	20190710; 20190710	no	yes	Jungclaus et al. (2019), Mauritsen et al. (2019), Müller et al. (2018), Schupfner et al. (2019)
MPI-ESM1-2-LR	Max-Planck-institute for Meteorology	r1i1p1f1	20190710; 20190710	no	yes	Mauritsen et al., (2019), Wieners et al., (2019a,b)
UKESM1-0-LL	Met Office Hadley	r1i1p1f2	20190627; 20190726	no	Yes, for crops and	Good et al., (2019),

	Center				pasture	Sellar et al., (2019), Tang et al., (2019)

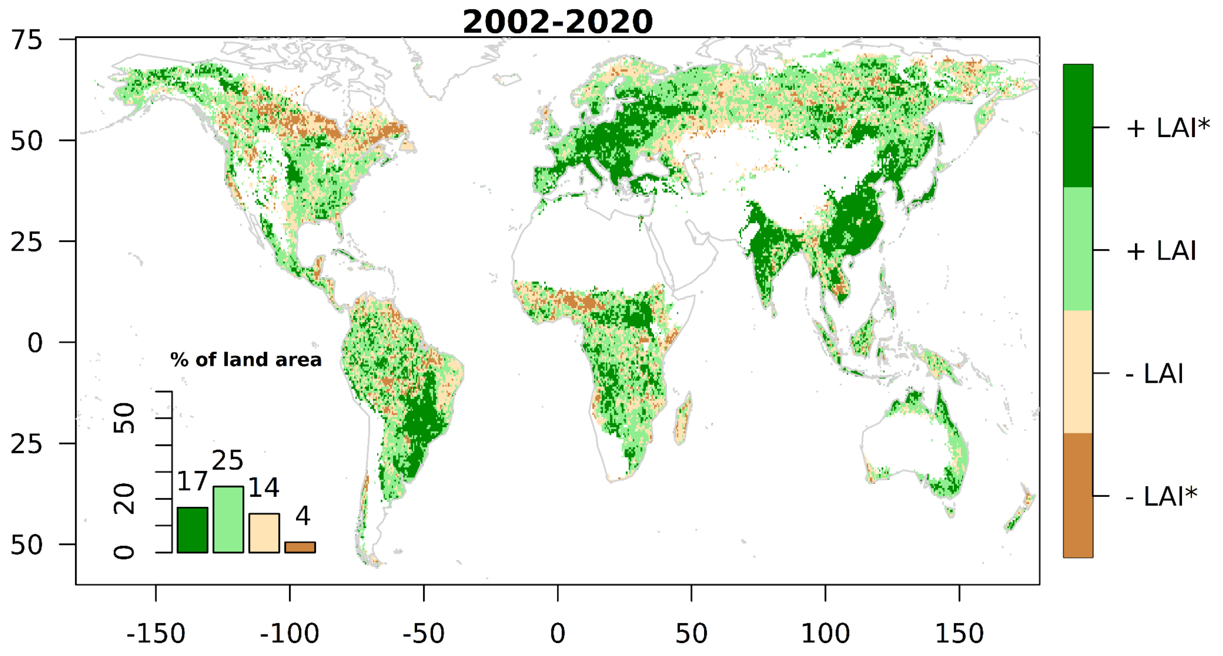

Figure S1: Similar to Figure 1b, but for MODIS.

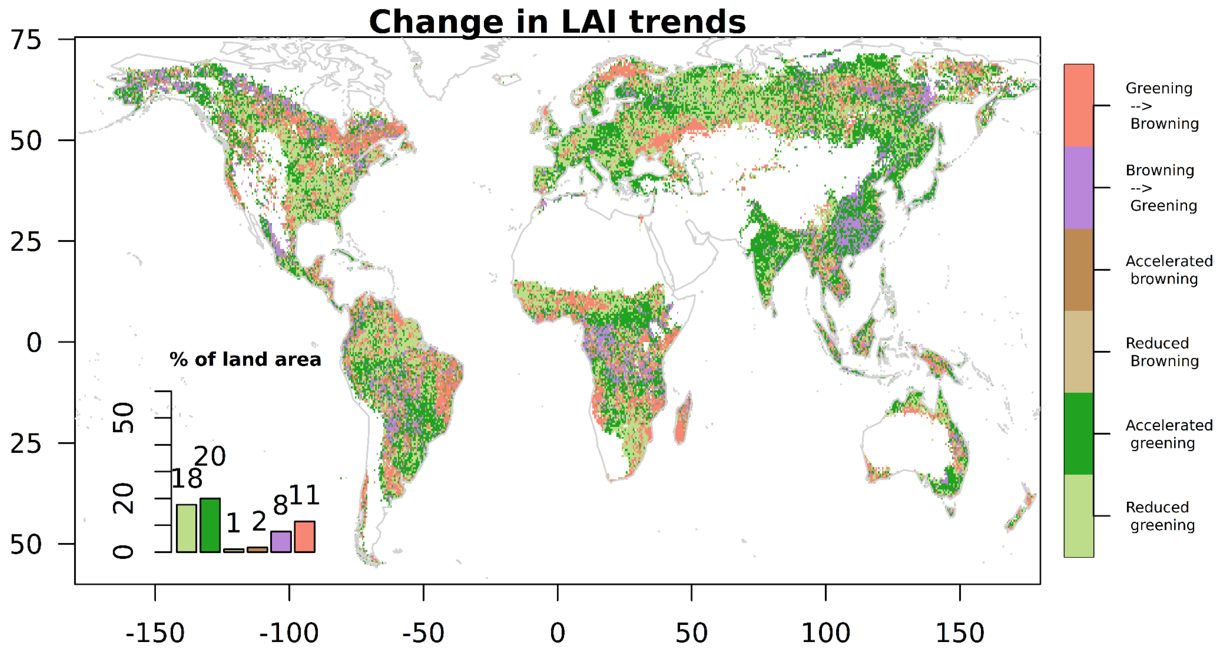

Figure S2: Change in observed LAI trends between 1982-2001 and 2002-2020.

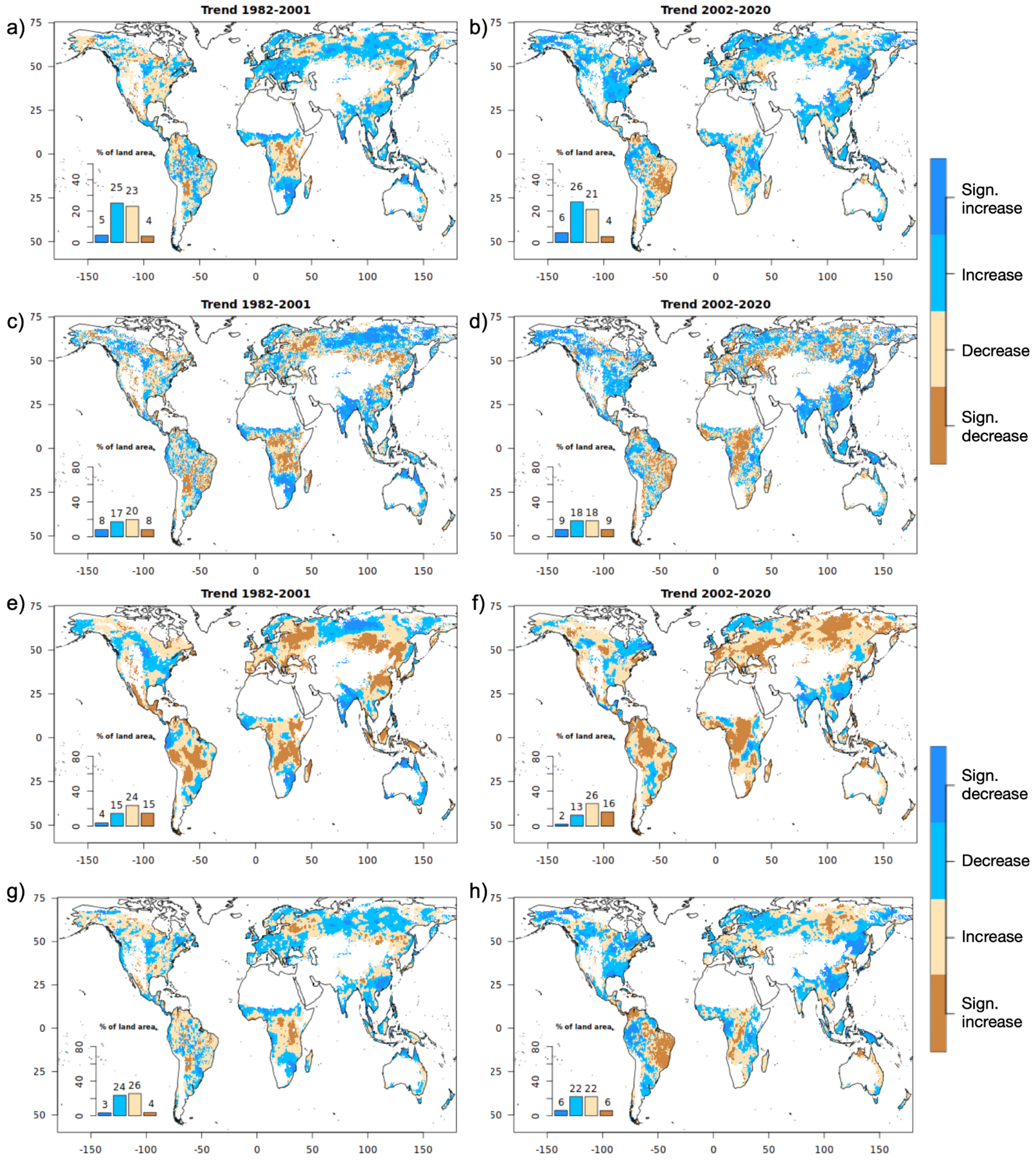

Figure S3: Trends of considered water-related variables during 1982-2001 and 2002-2020. Significance denoted at the 95% level by the asterisks. (a-b) Precipitation. (c-d) root zone soil moisture. (e-f) VPD. (g-h) Dryness index. All trends and their significance are determined with Mann-Kendall tests.

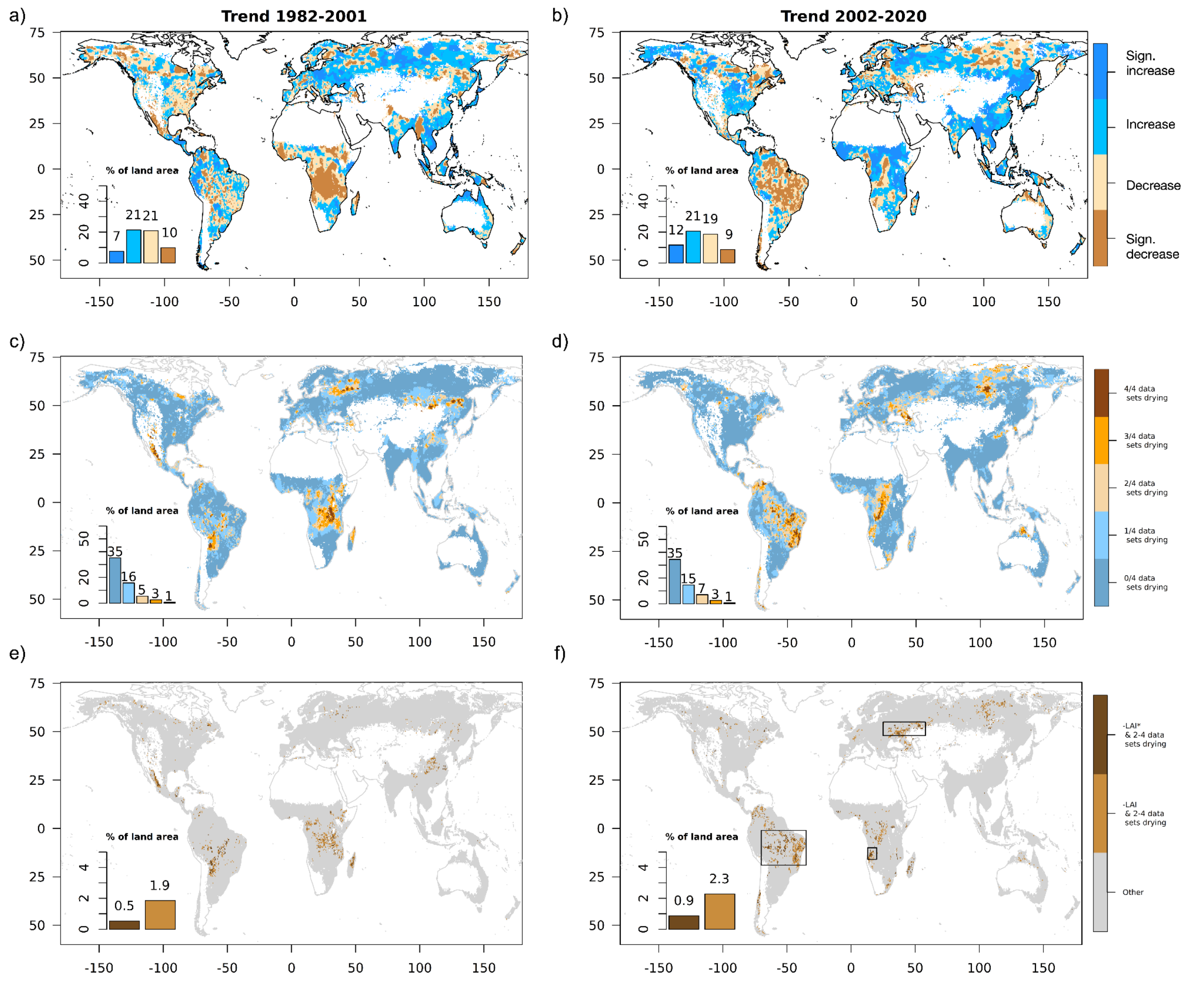

Fig S4: Repeating the analysis from Figure 1 with an alternative precipitation dataset. (a,b) Similar to Figure S3a,b but for precipitation data from CPC. (c-f) Similar to Figure 1c-f but with CPC precipitation data.

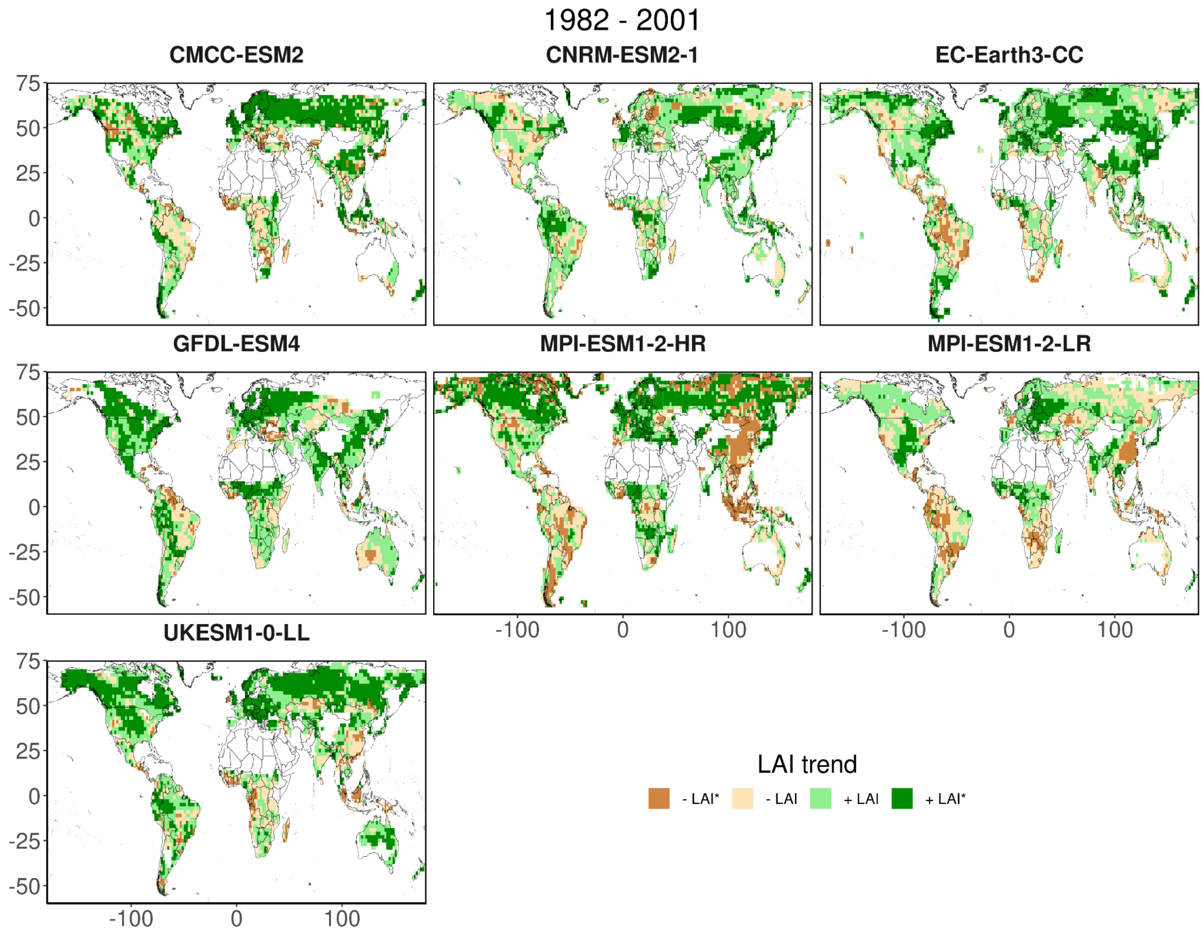

Figure S5: Similar to Figure 2a, but for individual Earth system models.

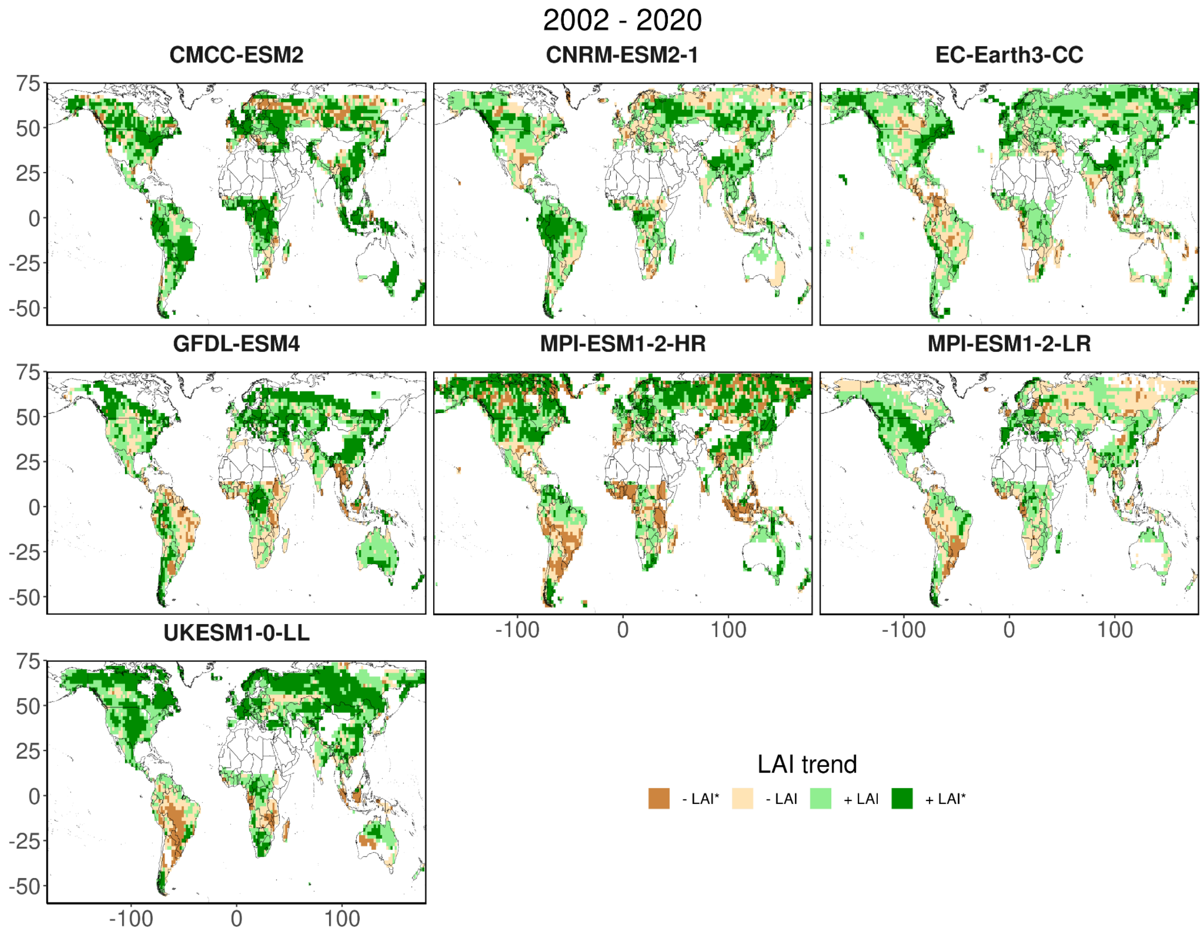

Figure S6: Similar to Figure 2b, but for individual Earth system models.

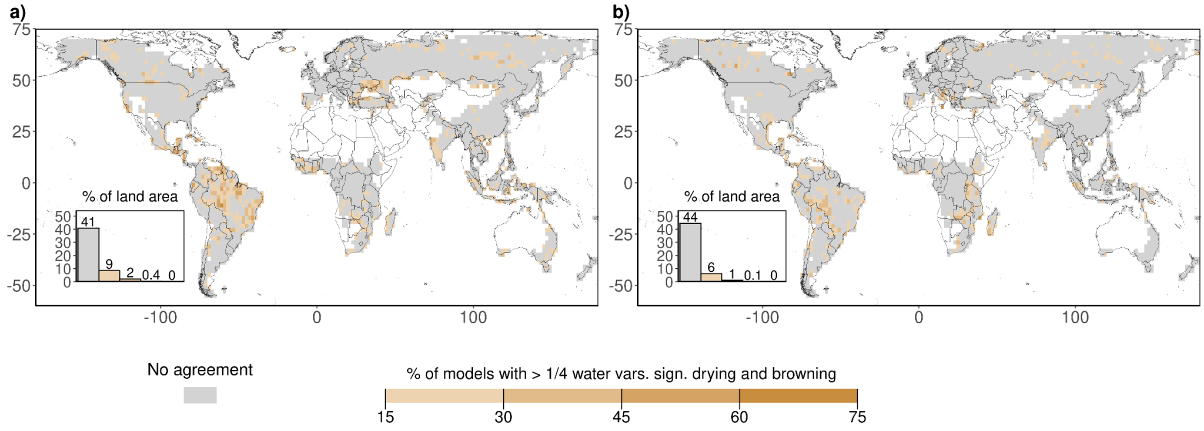

Fig. S7: Similar to Figure 2e-f, but also including areas with nonsignificant browning.

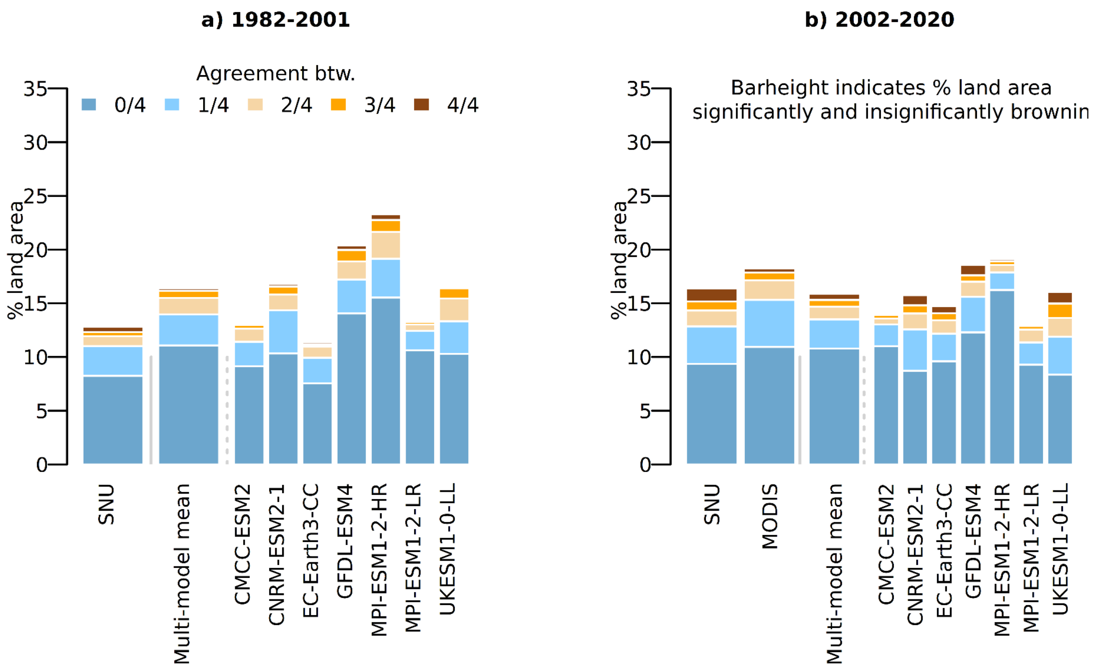

Figure S8. Similar to Figure 3, but additionally considering areas with insignificant browning.

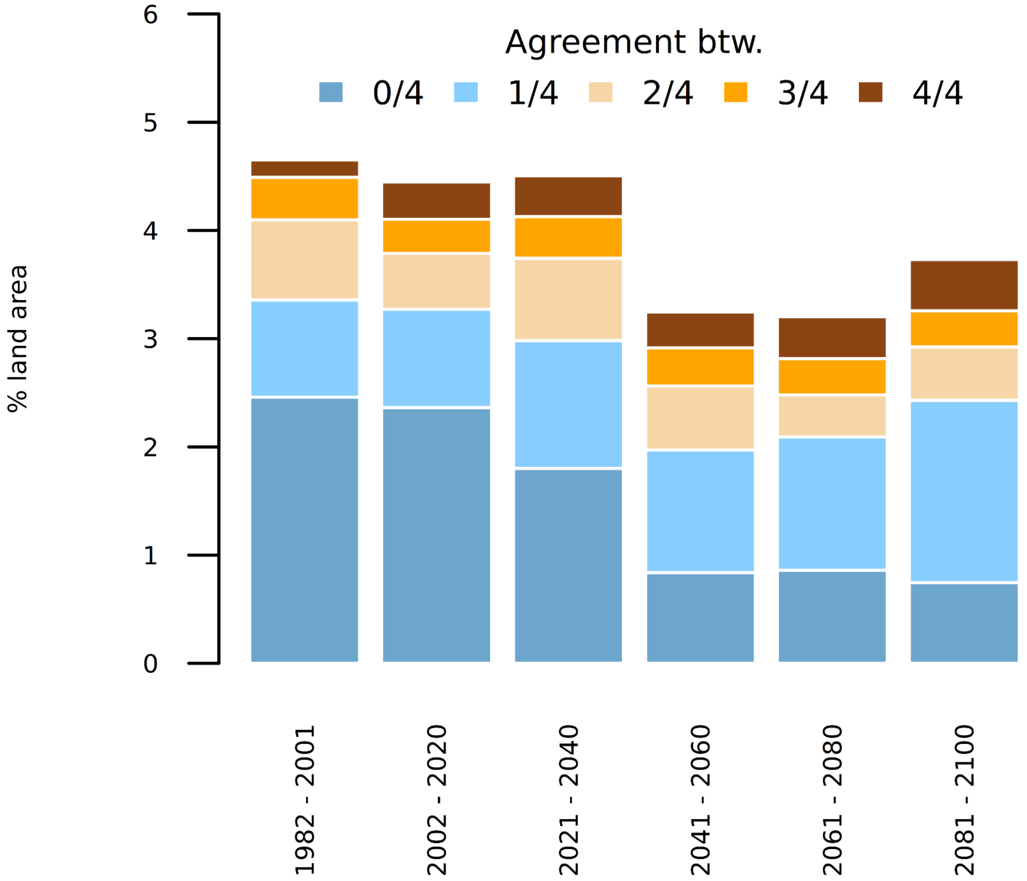

Figure S9. Similar to Figure 3, but for multi-model mean results for 20-year periods until the end of the century.

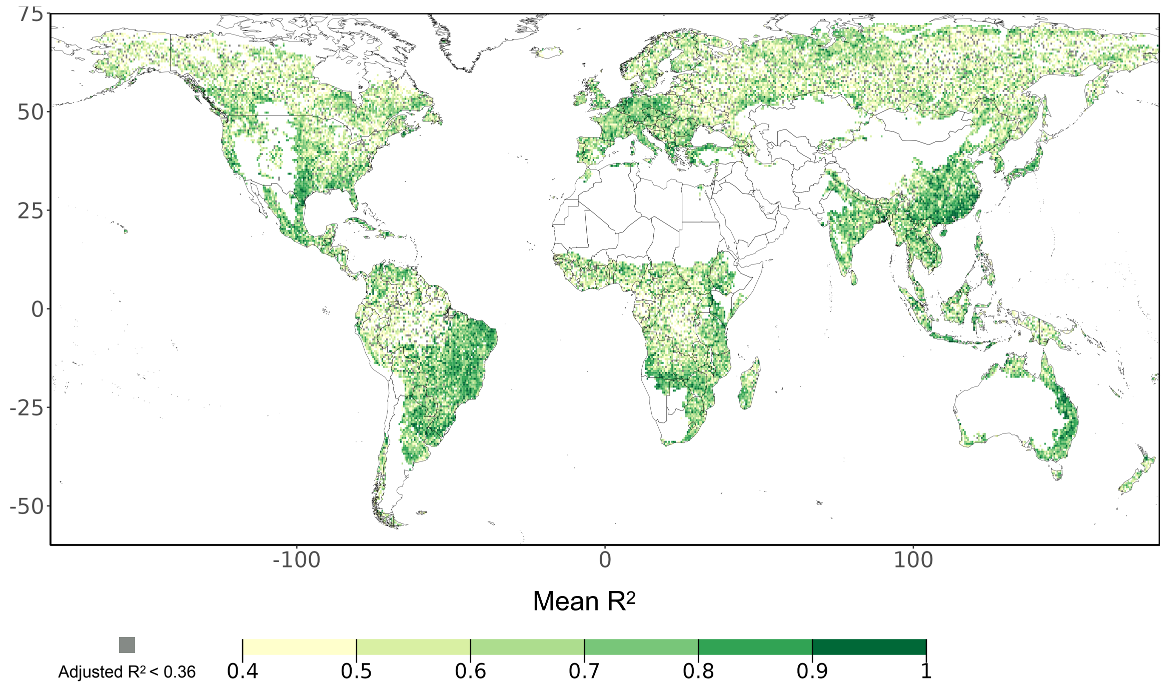

Fig. S10: Mean R^2 of all considered regression models at each grid cell in the observation-based analysis of drivers of LAI dynamics.

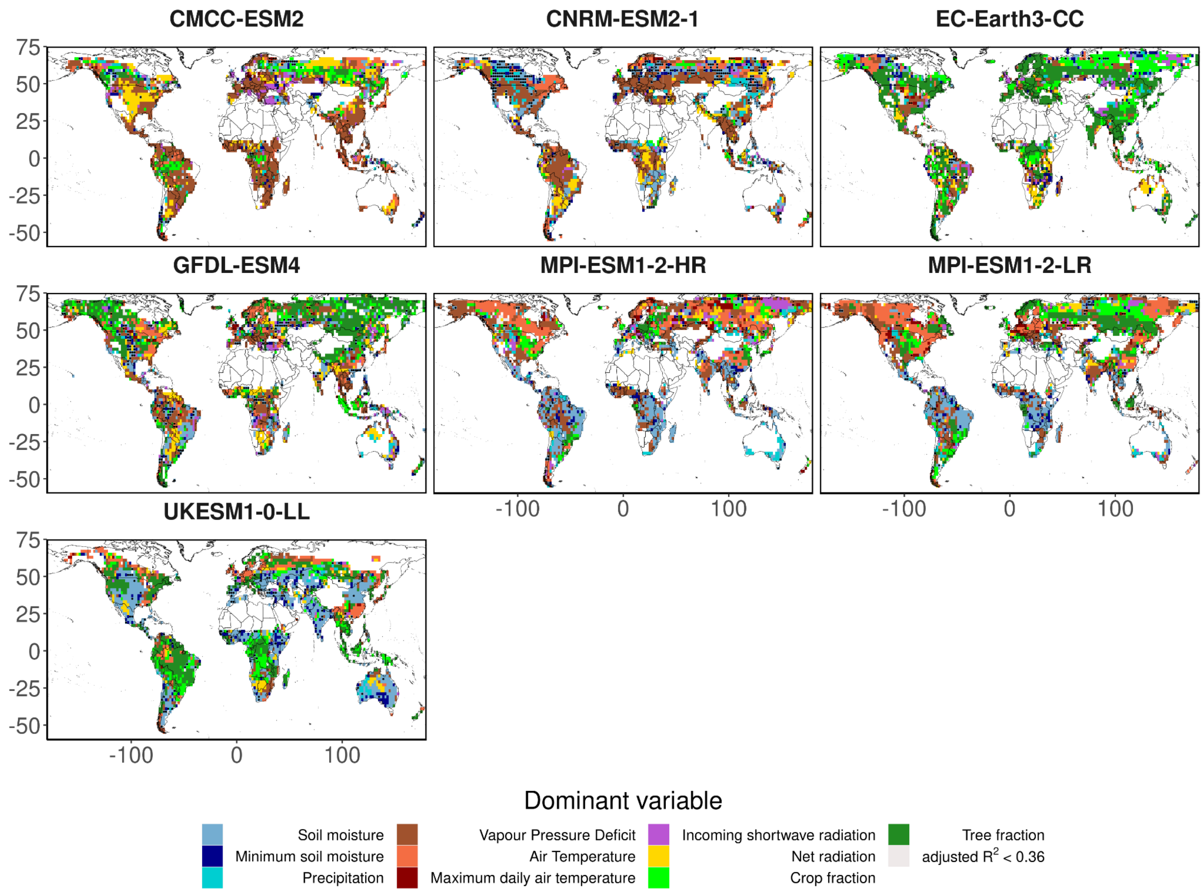

Figure S11: Similar to Figure 5a but for individual models.

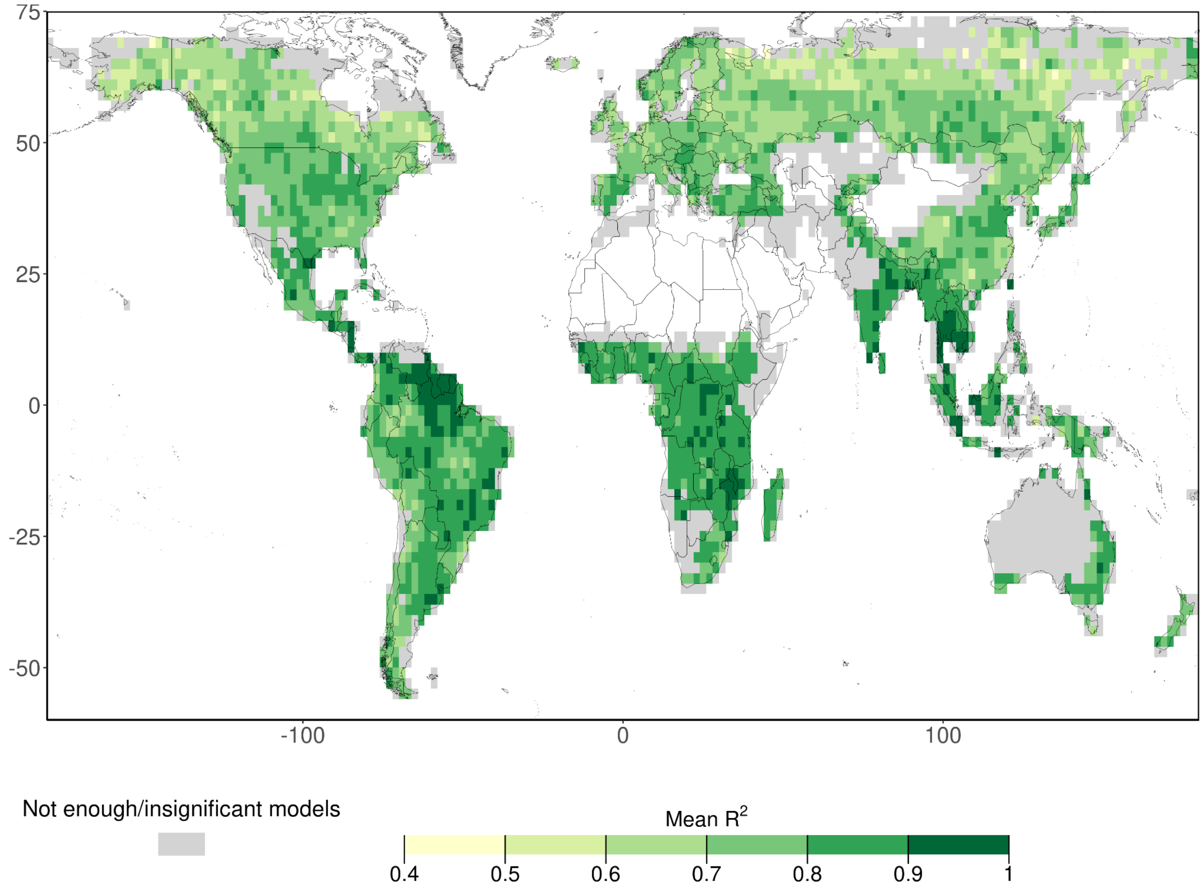

Fig. S12: Mean R^2 of all considered regression models, and across all considered Earth system models, at each grid cell in the model-based analysis of drivers of LAI dynamics.

References:

1. Jeong et al. 2024, doi: 10.1016/j.rse.2024.114282, https://www.sciencedirect.com/science/article/pii/S0034425724003006
2. Myneni et al. 2021, doi: 10.5067/MODIS/MOD15A2H.061, https://doi.org/10.5067/MODIS/MOD15A2H.061
3. Beck et al. 2019, doi: 10.1175/BAMS-D-17-0138.1, https://journals.ametsoc.org/view/journals/bams/100/3/bams-d-17-0138.1.xml
4. Chen et al. 2008, doi: 10.1029/2007jd009132, https://agupubs.onlinelibrary.wiley.com/doi/10.1029/2007JD009132
5. Miralles et al. 2025, doi: 10.1038/s41597-025-04610-y, https://www.nature.com/articles/s41597-025-04610-y
6. Defourny et al. 2017, maps.elie.ucl.ac.be/CCI/viewer/download/ESACCI-LC-Ph2-PUGv2_2.0.pdf
7. Hersbach et al. 2020, doi: 10.1002/qj.3803, https://rmets.onlinelibrary.wiley.com/doi/10.1002/qj.3803
8. Cherchi et al. 2019, doi: 10.1029/2018MS001369,https://agupubs.onlinelibrary.wiley.com/doi/full/10.1029/2018MS001369
9. Lovato and Peano 2020a, doi: 10.22033/ESGF/CMIP6.3825, https://doi.org/10.22033/ESGF/CMIP6.3825
10. Lovato and Peano 2020b, doi: 10.22033/ESGF/CMIP6.1365, https://doi.org/10.22033/ESGF/CMIP6.1365
11. Seferian 2018, doi: 10.22033/ESGF/CMIP6.4068, https://doi.org/10.22033/ESGF/CMIP6.4068

12. Seferian et al. 2019, doi: 10.1029/2019MS001791, https://doi.org/10.1029/2019MS001791
13. Voldoire 2019, doi: 10.22033/ESGF/CMIP6.4226, https://doi.org/10.22033/ESGF/CMIP6.4226
14. EC-Earth Consortium 2021a, doi: 10.22033/ESGF/CMIP6.4702, https://doi.org/10.22033/ESGF/CMIP6.4702
15. EC-Earth Consortium 2021b, doi: 10.22033/ESGF/CMIP6.15636, https://doi.org/10.22033/ESGF/CMIP6.15636
16. Döscher et al. 2022, doi: 10.5194/gmd-15-2973-2022, https://doi.org/10.5194/gmd-15-2973-2022
17. Dunne et al. 2020, doi: 10.1029/2019MS002015, https://doi.org/10.1029/2019MS002015
18. John et al. 2021, doi: 10.22033/ESGF/CMIP6.8706, https://doi.org/10.22033/ESGF/CMIP6.8706
19. Krasting et al. 2028, doi: 10.22033/ESGF/CMIP6.8597, https://doi.org/10.22033/ESGF/CMIP6.8597
20. Jungclaus et al. 2019, doi: 10.22033/ESGF/CMIP6.6594, https://doi.org/10.22033/ESGF/CMIP6.6594
21. Mauritzen et al. 2019, doi: 10.1029/2018MS001400, https://doi.org/10.1029/2018MS001400
22. Müller et al. 2018, doi: 10.1029/2017MS001217, https://doi.org/10.1029/2017MS001217
23. Schupfner et al. 2019, doi: 10.22033/ESGF/CMIP6.4403, https://doi.org/10.22033/ESGF/CMIP6.4403
24. Wieners et al. 2019a, doi: 10.22033/ESGF/CMIP6.6595, https://doi.org/10.22033/ESGF/CMIP6.6595
25. Wieners et al. 2019b, doi: 10.22033/ESGF/CMIP6.6705, https://doi.org/10.22033/ESGF/CMIP6.6705
26. Tang et al. 2019, doi: 10.22033/ESGF/CMIP6.6113, https://doi.org/10.22033/ESGF/CMIP6.6113
27. Sellar et al. 2019, doi: 10.1029/2019MS001739, https://doi.org/10.1029/2019MS001739
28. Good et al. 2019, doi: 10.22033/ESGF/CMIP6.10901, https://doi.org/10.22033/ESGF/CMIP6.10901
